# Crystal structure of (−)-(2*R*,3*S*,4*R*,5*R*)-5-(1,3-di­thian-2-yl)-3-methyl-1-(triiso­propyl­sil­yloxy)hexane-2,4-diol

**DOI:** 10.1107/S160053681402443X

**Published:** 2014-11-21

**Authors:** Alejandra Cruz-Montanez, Dalice M. Piñero Cruz, Jose A. Prieto

**Affiliations:** a University of Puerto Rico, Rio Piedras Campus, Department of Chemistry, PO Box 23346, San Juan, 000936-8377, Puerto Rico

**Keywords:** crystal structure, polypropionate, stereo­tetra­ds, 1,3-di­thiane

## Abstract

The title compound, C_20_H_42_O_3_S_2_Si, crystallized with two independent mol­ecules (*A* and *B*) in the asymmetric unit. They consist of *syn,anti,anti*-stereo­tetrads with a 1,3-di­thiane motif and a primary alcohol protected as the triisopropyl silyl ether. The 1,3-di­thiane ring adopts a chair conformation, while the rest of each mol­ecule displays a common zigzag conformation. There is an intra­molecular O—H⋯O hydrogen bond in each mol­ecule. In the crystal, the *A* and *B* mol­ecules are linked *via* O—H⋯O hydrogen bonds, forming –*A*–*B*–*A-*-*B-*- chains along [010]. The absolute structure was determined by resonant scattering (anomalous scattering) [Flack parameter = 0.035 (8)].

## Related literature   

The title compound was obtained as part of our studies toward the synthesis of (+)-crocacin C, using an epoxide-based approach for the stereo­tetrad construction. For the one- and two-dimensional NMR spectra of the acetonide product, see: Rychnovsky & Skalitzky (1990 ▶). For the isolation and bio­logical activity of crocacin, see: Kunze *et al.* (1994 ▶); Jansen *et al.* (1999 ▶). For the di­thiane epoxide cleavage, see: Ide & Nakata (1999 ▶); Ide *et al.* (1999 ▶). For polypropionate-related synthesis and background, see: Li & Menche (2009 ▶); Rodríguez-Berríos *et al.* (2011 ▶); Torres *et al.* (2009 ▶); Dávila *et al.* (2007 ▶); Rodríguez *et al.* (2006 ▶). For biological activities of polypropionates, see; Li & Menche (2009 ▶); Rohr (2000 ▶). For a related structure, see: Valentín *et al.* (2012 ▶).
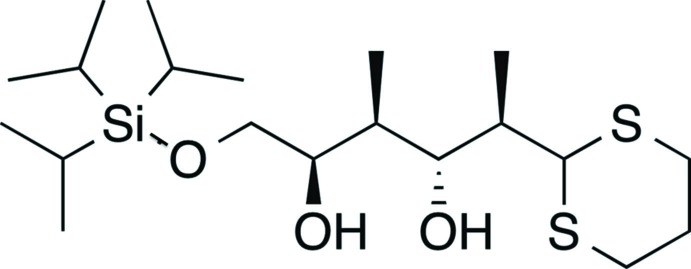


## Experimental   

### Crystal data  

C_20_H_42_O_3_S_2_Si
*M* *_r_* = 422.74
Monoclinic, 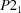

*a* = 15.9691 (4) Å
*b* = 8.3420 (2) Å
*c* = 19.1245 (5) Å
β = 101.253 (2)°
*V* = 2498.68 (11) Å^3^
*Z* = 4
Cu *K*α radiation
μ = 2.51 mm^−1^
*T* = 124 K
0.10 × 0.05 × 0.05 mm

### Data collection  

Bruker APEXII CCD diffractometer
Absorption correction: multi-scan (*SADABS*; Sheldrick, 1996 ▶) *T*_min_ = 0.860, *T*_max_ = 0.882
38635 measured reflections
9653 independent reflections
8808 reflections with *I* > 2σ(*I*)
*R*_int_ = 0.062

### Refinement  

*R*[*F* ^2^ > 2σ(*F* ^2^)] = 0.036
*wR*(*F* ^2^) = 0.088
*S* = 1.02
9653 reflections
501 parameters
1 restraint
H atoms treated by a mixture of independent and constrained refinement
Δρ_max_ = 0.41 e Å^−3^
Δρ_min_ = −0.18 e Å^−3^
Absolute structure: Flack *x* determined using 3771 quotients [(*I* ^+^)−(*I* ^−^)]/[(*I* ^+^)+(*I* ^−^)] (Parsons *et al.*, 2013 ▶)
Absolute structure parameter: 0.035 (8)

### 

Data collection: *APEX2* (Bruker, 2012 ▶); cell refinement: *SAINT* (Bruker, 2012 ▶); data reduction: *SAINT*; program(s) used to solve structure: *SHELXS97* (Sheldrick, 2008 ▶); program(s) used to refine structure: *SHELXL2014* (Sheldrick, 2008 ▶); molecular graphics: *Mercury* (Macrae *et al.*, 2008 ▶); software used to prepare material for publication: *SHELXTL* (Sheldrick, 2008 ▶).

## Supplementary Material

Crystal structure: contains datablock(s) I, New_Global_Publ_Block. DOI: 10.1107/S160053681402443X/su5013sup1.cif

Structure factors: contains datablock(s) I. DOI: 10.1107/S160053681402443X/su5013Isup2.hkl

Supporting information file. DOI: 10.1107/S160053681402443X/su5013Isup3.cml

**Fig. 1.**
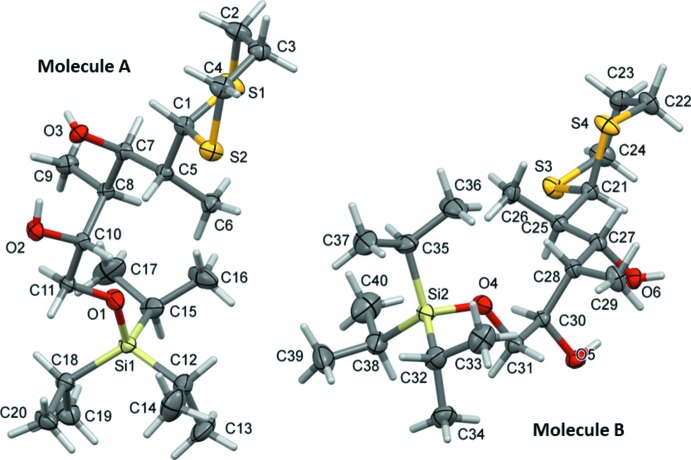
A view of the molecular structure of the two independent molecules (A and B) of the title compound, showing the atom labelling. Displacement ellipsoids are drawn at the 50% probability level.

**Fig. 2.**
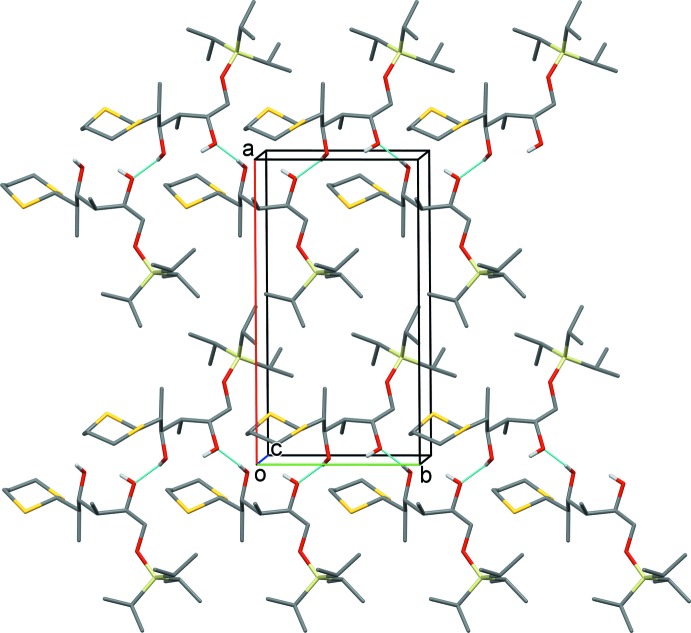
A view along the c axis of the crystal packing of the title compound. The intermolecular hydrogen bonds are shown as dashed lines (see Table 1 for details; H atoms not involved in these interactions have been omitted for clarity).

A B . DOI: 10.1107/S160053681402443X/su5013fig1.tif
A view of the mol­ecular structure of the two independent mol­ecules (*A* and *B*) of the title compound, showing the atom labelling. Displacement ellipsoids are drawn at the 50% probability level.

c . DOI: 10.1107/S160053681402443X/su5013fig2.tif
A view along the *c* axis of the crystal packing of the title compound. The inter­molecular hydrogen bonds are shown as dashed lines (see Table 1 for details; H atoms not involved in these inter­actions have been omitted for clarity).

**Fig. 3.**
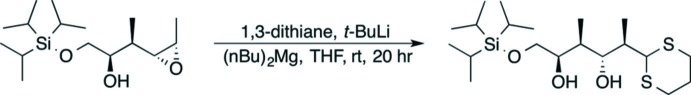
Reaction scheme.

. DOI: 10.1107/S160053681402443X/su5013fig3.tif
Reaction scheme.

CCDC reference: 1029553

Additional supporting information:  crystallographic information; 3D view; checkCIF report

## Figures and Tables

**Table 1:** Hydrogen-bond geometry (, )

*D*H*A*	*D*H	H*A*	*D* *A*	*D*H*A*
O2H100O3	0.76(4)	2.00(4)	2.705(4)	154(5)
O5H103O6	0.81(5)	1.94(5)	2.689(3)	153(5)
O3H101O5^i^	0.66(3)	2.11(4)	2.751(3)	167(5)
O6H102O2^ii^	0.76(4)	1.93(4)	2.685(3)	175(4)

Symmetry codes: (i) 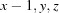; (ii) 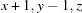.

## supplementary crystallographic information

### S1. Comment

The synthesis of the title compound was obtained through regioselective epoxide cleavage of (-)-(2*R*,3*R*)-3-((2*R*,3*S*)-3-methyloxiran-2-yl)-1- ((triisopropylsilyl)oxy)butan-2-ol with 1,3-dithiane in the presence of *tert-*butyllithium and di-*n*-butylmagnesium as metallation reagents. This reaction afforded the optically active *syn,anti,anti*-polypropionate unit needed for the synthesis of (+)-crocacin C with the correct 2*R*,3*S*,4*R*,5*R* absolute configuration. The resulting stereochemistry was confirmed by 1D- and 2D-NMR spectra of the acetonide product (Rychnovsky & Skalitzky, 1990), as well as by X-ray crystallography.

Polypropionates are a common moiety consisting of a stereodefined array of methyl and hydroxy substituents in an aliphatic chain. Their structure is found in various natural products, many of them possessing a wide range of biological activity (typically antibiotic, antitumor, antifungal, antiparasitic, among others) (Rohr, 2000). Different methodologies have been applied for their synthesis, although the aldol approach continues to be one of the most used methods (Li & Menche, 2009). An alternative approach to their synthesis is the regioselective cleavage of oxirane rings. The methodology developed in our lab consists of a reiterative sequence in which a disubstituted epoxide is regioselectively cleaved with either a propynylaluminum reagent (Dávila *et al.*, 2007) or Grignard reagent (Rodríguez *et al.*, 2006), followed by reduction (if needed), and further epoxidation of the newly formed alkene. In this methodology, the configuration of the hydroxyl functionality is determined by the configuration of the epoxide, while the *syn/anti* relative configuration of the methyl and hydroxyl groups is defined by the epoxide geometry. In this substrate controlled synthesis of the title compound, the configuration of the formed hydroxyl group was determined by the configuration of the substrate epoxide, while the *anti* relative configuration obtained between the formed hydroxyl and methyl group was due to the *cis*-geometry of the epoxide.

### S2. Experimental

The synthesis of the title compound is illustrated in Fig. 3. It was synthesized using Nakata's modified protocol (Ide *et al.*, 1999). To a flame-dried round bottom flask was added 12 mL of *t-*butyllithium (19.8 mmol, 1.7 M in pentane) and 4.95 mL of di-*n*-butylmagnesium (4.95 mmol, 1.0 M in heptane) at rt. The mixed reagents were then transferred via cannula to another flame-dried round bottom flask containing a stirred solution of 1,3-dithiane (0.99 g, 8.25 mmol) in dry THF (16 mL) at rt. The reaction mixture turned bright yellow, and was left stirring for 1 hr after which the epoxide was added (1.0 g, 3.30 mmol). After 20 h at rt, the reaction was quenched by adding saturated aqueous NH_4_Cl, and the resulting mixture was extracted with ethyl acetate. The combined organic phase was washed with brine, dried over MgSO_4_, and concentrated under reduced pressure. The resulting dithiane was purified using column chromatography (9:1 hexane/Et_2_O), yielding 0.649 g (66%) of the dithiane product as a white solid, m.p.: 367-369 K. Colourless crystals, suitable for X-ray diffraction, were obtained by slow diffusion of diethylether into a solution in hexanes at room temperature over a period of 2 days. ^1^H NMR (500 MHz, CDCl_3_) δ 4.76 (d, *J* = 2.6 Hz, 1H), 4.05 (dddd, *J* = 7.4, 5.5, 1.9, 1.9 Hz, 1H), 3.67 (dd, *J* = 9.6, 7.6 Hz, 1H), 3.60 (ddd, *J* = 10.1, 7.8, 2.8 Hz, 1H), 3.56 (dd, *J* = 9.6, 5.5 Hz, 1H), 3.32 (d, *J* = 7.9 Hz, 1H), 3.04 (ddd, *J* = 13.8, 12.6, 2.5 Hz, 1H), 2.95 – 2.80 (m, 3H), 2.85 (d, *J* = 7.4 Hz, 1H), 2.15 (m, 1H), 2.11 (m, 1H), 1.88 (m, 1H), 1.84 (m, 1H), 1.08 (d, *J* = 7.1 Hz , 3H), 1.05 (m, 21H), 1.02 (d, *J* = 6.9 Hz, 3H). ^13^C NMR (125 MHz, CDCl_3_) δ 77.7, 71.4, 65.2, 52.4, 42.6, 33.9, 31.5, 30.8, 26.5, 17.9, 13.1, 11.9, 11.5. [α]^20^_D_= -12.9 (c = 1.0, CHCl_3_). Anal. Calcd. for C_20_H_42_O_3_S_2_Si: C, 56.82 %, H, 10.01 %; Found: C, 56.53 %, H, 9.78 %.

### S3. Refinement

All atoms, except hydrogen, were refined anisotropically. The H atoms were placed at calculated positions using suitable riding models except those located on the hydroxy groups, which were found directly on the difference Fourier map and refined using DFIX constraints. Aliphatic H atoms were included in geometrically calculated positions, with C—H distances constrained to 0.98–1.00 Å. Methyl H atoms displacement parameters were set at *U*_iso_(H) = 1.5*U*_eq_(C). Hydroxy H atoms were located from a difference Fourier map and allowed to refine freely.

### Crystal data

**Table:**

C_20_H_42_O_3_S_2_Si	*F*(000) = 928
*M**_r_* = 422.74	*D*_x_ = 1.124 Mg m^−^^3^
Monoclinic, *P*2_1_	Cu *K*α radiation, λ = 1.54178 Å
*a* = 15.9691 (4) Å	Cell parameters from 8415 reflections
*b* = 8.3420 (2) Å	θ = 2.8–70.9°
*c* = 19.1245 (5) Å	µ = 2.51 mm^−^^1^
β = 101.253 (2)°	*T* = 124 K
*V* = 2498.68 (11) Å^3^	Block, colourless
*Z* = 4	0.10 × 0.05 × 0.05 mm

### Data collection

**Table:**

Bruker APEXII CCD diffractometer	9653 independent reflections
Radiation source: sealed tube	8808 reflections with *I* > 2σ(*I*)
Graphite monochromator	*R*_int_ = 0.062
φ and ω scans	θ_max_ = 71.8°, θ_min_ = 2.4°
Absorption correction: multi-scan (*SADABS*; Sheldrick, 1996)	*h* = −19→19
*T*_min_ = 0.860, *T*_max_ = 0.882	*k* = −10→10
38635 measured reflections	*l* = −23→23

### Refinement

**Table:**

Refinement on *F*^2^	Secondary atom site location: difference Fourier map
Least-squares matrix: full	Hydrogen site location: mixed
*R*[*F*^2^ > 2σ(*F*^2^)] = 0.036	H atoms treated by a mixture of independent and constrained refinement
*wR*(*F*^2^) = 0.088	*w* = 1/[σ^2^(*F*_o_^2^) + (0.0453*P*)^2^] where *P* = (*F*_o_^2^ + 2*F*_c_^2^)/3
*S* = 1.02	(Δ/σ)_max_ = 0.001
9653 reflections	Δρ_max_ = 0.41 e Å^−^^3^
501 parameters	Δρ_min_ = −0.18 e Å^−^^3^
1 restraint	Absolute structure: Flack *x* determined using 3771 quotients [(*I*^+^)-(*I*^-^)]/[(*I*^+^)+(*I*^-^)] (Parsons *et al.*, 2013)
Primary atom site location: structure-invariant direct methods	Absolute structure parameter: 0.035 (8)

### Special details

**Table:**

Geometry. All e.s.d.'s (except the e.s.d. in the dihedral angle between two l.s. planes) are estimated using the full covariance matrix. The cell e.s.d.'s are taken into account individually in the estimation of e.s.d.'s in distances, angles and torsion angles; correlations between e.s.d.'s in cell parameters are only used when they are defined by crystal symmetry. An approximate (isotropic) treatment of cell e.s.d.'s is used for estimating e.s.d.'s involving l.s. planes.

### Fractional atomic coordinates and isotropic or equivalent isotropic displacement parameters (Å^2^)

**Table:**

	*x*	*y*	*z*	*U*_iso_*/*U*_eq_	
S1	0.16186 (6)	0.07437 (9)	0.13813 (4)	0.03371 (19)	
S2	0.12503 (5)	0.30070 (9)	0.01309 (4)	0.02892 (17)	
Si1	0.34129 (5)	0.86798 (10)	0.37531 (4)	0.02346 (17)	
O1	0.26097 (14)	0.7796 (3)	0.32190 (13)	0.0346 (5)	
O2	0.04565 (13)	0.7418 (3)	0.22348 (11)	0.0246 (4)	
O3	0.01352 (14)	0.4290 (3)	0.19173 (11)	0.0231 (4)	
C1	0.11141 (19)	0.2611 (4)	0.10346 (14)	0.0228 (6)	
H1	0.0488	0.2513	0.1026	0.027*	
C2	0.0977 (2)	−0.0623 (4)	0.07562 (17)	0.0341 (7)	
H2A	0.1158	−0.1736	0.0886	0.041*	
H2B	0.0372	−0.0516	0.0799	0.041*	
C3	0.1048 (2)	−0.0325 (4)	−0.00130 (16)	0.0312 (7)	
H3A	0.1658	−0.0371	−0.0050	0.037*	
H3B	0.0746	−0.1193	−0.0314	0.037*	
C4	0.0684 (2)	0.1269 (4)	−0.02988 (16)	0.0326 (7)	
H4A	0.0081	0.1329	−0.0243	0.039*	
H4B	0.0688	0.1317	−0.0815	0.039*	
C5	0.14528 (18)	0.4037 (3)	0.15198 (14)	0.0205 (6)	
H5	0.1256	0.5039	0.1250	0.025*	
C6	0.24314 (19)	0.4086 (4)	0.16968 (16)	0.0284 (6)	
H6A	0.2620	0.5113	0.1922	0.043*	
H6B	0.2654	0.3968	0.1257	0.043*	
H6C	0.2645	0.3208	0.2024	0.043*	
C7	0.10345 (17)	0.3999 (3)	0.21755 (14)	0.0200 (6)	
H7	0.1103	0.2897	0.2385	0.024*	
C8	0.13915 (17)	0.5211 (3)	0.27627 (13)	0.0188 (5)	
H8	0.2010	0.4961	0.2932	0.023*	
C9	0.0945 (2)	0.4992 (4)	0.33964 (15)	0.0267 (6)	
H9A	0.0348	0.5332	0.3260	0.040*	
H9B	0.1234	0.5642	0.3798	0.040*	
H9C	0.0967	0.3860	0.3536	0.040*	
C10	0.13291 (17)	0.6938 (3)	0.24864 (14)	0.0189 (5)	
H10	0.1632	0.6998	0.2076	0.023*	
C11	0.17329 (18)	0.8160 (3)	0.30402 (15)	0.0229 (6)	
H11A	0.1648	0.9258	0.2843	0.028*	
H11B	0.1471	0.8092	0.3468	0.028*	
C12	0.4239 (2)	0.8970 (4)	0.31910 (18)	0.0331 (7)	
H12	0.4404	0.7863	0.3070	0.040*	
C13	0.5064 (2)	0.9745 (6)	0.3577 (2)	0.0504 (10)	
H13A	0.5476	0.9778	0.3258	0.076*	
H13B	0.5301	0.9117	0.4002	0.076*	
H13C	0.4945	1.0839	0.3717	0.076*	
C14	0.3906 (3)	0.9783 (6)	0.2472 (2)	0.0548 (11)	
H14A	0.3811	1.0925	0.2548	0.082*	
H14B	0.3366	0.9283	0.2243	0.082*	
H14C	0.4326	0.9661	0.2165	0.082*	
C15	0.3824 (2)	0.7206 (4)	0.44862 (19)	0.0364 (8)	
H15	0.4322	0.7713	0.4808	0.044*	
C16	0.4144 (3)	0.5676 (5)	0.4182 (3)	0.0536 (11)	
H16A	0.4326	0.4904	0.4568	0.080*	
H16B	0.4627	0.5935	0.3956	0.080*	
H16C	0.3682	0.5209	0.3827	0.080*	
C17	0.3148 (3)	0.6832 (6)	0.4934 (3)	0.0612 (14)	
H17A	0.2650	0.6338	0.4631	0.092*	
H17B	0.2976	0.7828	0.5139	0.092*	
H17C	0.3389	0.6092	0.5320	0.092*	
C18	0.30313 (19)	1.0552 (4)	0.41417 (16)	0.0265 (6)	
H18	0.2490	1.0257	0.4298	0.032*	
C19	0.3641 (2)	1.1173 (5)	0.48091 (19)	0.0394 (8)	
H19A	0.4177	1.1520	0.4680	0.059*	
H19B	0.3758	1.0315	0.5164	0.059*	
H19C	0.3377	1.2082	0.5009	0.059*	
C20	0.2804 (2)	1.1928 (4)	0.36050 (19)	0.0357 (8)	
H20A	0.2537	1.2801	0.3826	0.054*	
H20B	0.2406	1.1539	0.3183	0.054*	
H20C	0.3325	1.2323	0.3464	0.054*	
S3	0.86997 (5)	−0.22386 (9)	0.49223 (4)	0.02930 (18)	
S4	0.83329 (5)	−0.44672 (9)	0.36602 (4)	0.03153 (18)	
Si2	0.62445 (5)	0.36336 (10)	0.15228 (4)	0.02448 (17)	
O4	0.70944 (13)	0.2498 (3)	0.17766 (11)	0.0284 (5)	
O5	0.92962 (13)	0.2292 (3)	0.26956 (12)	0.0265 (4)	
O6	0.96444 (13)	−0.0785 (3)	0.30587 (11)	0.0236 (4)	
C21	0.87904 (18)	−0.2564 (3)	0.40019 (14)	0.0214 (6)	
H21	0.9412	−0.2589	0.3985	0.026*	
C22	0.9035 (2)	−0.5798 (4)	0.42560 (16)	0.0311 (7)	
H22A	0.8880	−0.6921	0.4121	0.037*	
H22B	0.9629	−0.5623	0.4194	0.037*	
C23	0.8996 (2)	−0.5564 (4)	0.50376 (16)	0.0281 (6)	
H23A	0.9340	−0.6412	0.5322	0.034*	
H23B	0.8398	−0.5690	0.5097	0.034*	
C24	0.9324 (2)	−0.3933 (4)	0.53254 (16)	0.0325 (7)	
H24A	0.9918	−0.3803	0.5253	0.039*	
H24B	0.9340	−0.3912	0.5845	0.039*	
C25	0.83857 (17)	−0.1149 (3)	0.35346 (14)	0.0210 (6)	
H25	0.8580	−0.0140	0.3799	0.025*	
C26	0.74090 (18)	−0.1173 (4)	0.34094 (16)	0.0286 (6)	
H26A	0.7185	−0.0135	0.3218	0.043*	
H26B	0.7227	−0.1377	0.3862	0.043*	
H26C	0.7190	−0.2022	0.3068	0.043*	
C27	0.87422 (17)	−0.1132 (3)	0.28443 (14)	0.0208 (6)	
H27	0.8682	−0.2235	0.2636	0.025*	
C28	0.83230 (18)	0.0047 (3)	0.22688 (14)	0.0203 (5)	
H28	0.7698	−0.0201	0.2160	0.024*	
C29	0.8661 (2)	−0.0222 (4)	0.15776 (15)	0.0312 (7)	
H29A	0.9259	0.0117	0.1649	0.047*	
H29B	0.8320	0.0407	0.1191	0.047*	
H29C	0.8619	−0.1362	0.1452	0.047*	
C30	0.84170 (17)	0.1791 (3)	0.25221 (14)	0.0198 (5)	
H30	0.8171	0.1882	0.2963	0.024*	
C31	0.79599 (18)	0.2985 (4)	0.19808 (15)	0.0234 (6)	
H31A	0.7991	0.4072	0.2193	0.028*	
H31B	0.8233	0.3015	0.1559	0.028*	
C32	0.6343 (2)	0.4814 (4)	0.07042 (18)	0.0355 (7)	
H32	0.5776	0.5324	0.0519	0.043*	
C33	0.6545 (2)	0.3703 (6)	0.01114 (17)	0.0457 (9)	
H33A	0.6638	0.4353	−0.0294	0.069*	
H33B	0.6065	0.2972	−0.0045	0.069*	
H33C	0.7061	0.3080	0.0297	0.069*	
C34	0.7010 (2)	0.6176 (5)	0.0870 (2)	0.0431 (9)	
H34A	0.7575	0.5714	0.1051	0.065*	
H34B	0.6854	0.6893	0.1230	0.065*	
H34C	0.7022	0.6783	0.0433	0.065*	
C35	0.5335 (2)	0.2169 (4)	0.13596 (18)	0.0343 (7)	
H35	0.5193	0.1932	0.1836	0.041*	
C36	0.5558 (2)	0.0553 (5)	0.1054 (2)	0.0487 (10)	
H36A	0.5071	−0.0178	0.1017	0.073*	
H36B	0.6055	0.0086	0.1371	0.073*	
H36C	0.5690	0.0721	0.0580	0.073*	
C37	0.4521 (2)	0.2851 (5)	0.0896 (2)	0.0523 (11)	
H37A	0.4609	0.2991	0.0406	0.078*	
H37B	0.4388	0.3890	0.1087	0.078*	
H37C	0.4046	0.2109	0.0897	0.078*	
C38	0.6164 (2)	0.5038 (5)	0.2277 (2)	0.0392 (8)	
H38	0.6716	0.5636	0.2388	0.047*	
C39	0.5455 (3)	0.6314 (6)	0.2079 (3)	0.0607 (12)	
H39A	0.4894	0.5796	0.2019	0.091*	
H39B	0.5512	0.6840	0.1633	0.091*	
H39C	0.5508	0.7115	0.2461	0.091*	
C40	0.6076 (3)	0.4152 (6)	0.2961 (2)	0.0589 (12)	
H40A	0.6094	0.4927	0.3349	0.088*	
H40B	0.6547	0.3387	0.3089	0.088*	
H40C	0.5531	0.3574	0.2883	0.088*	
H101	−0.007 (2)	0.392 (5)	0.2137 (18)	0.018 (10)*	
H102	0.988 (2)	−0.124 (5)	0.2815 (19)	0.030 (10)*	
H100	0.022 (3)	0.665 (5)	0.210 (2)	0.028 (10)*	
H103	0.955 (3)	0.150 (6)	0.286 (2)	0.041 (12)*	

### Atomic displacement parameters (Å^2^)

**Table:**

	*U*^11^	*U*^22^	*U*^33^	*U*^12^	*U*^13^	*U*^23^
S1	0.0478 (5)	0.0229 (4)	0.0248 (4)	0.0026 (3)	−0.0070 (3)	0.0004 (3)
S2	0.0402 (4)	0.0279 (4)	0.0189 (3)	−0.0032 (3)	0.0062 (3)	0.0004 (3)
Si1	0.0160 (3)	0.0193 (4)	0.0325 (4)	−0.0007 (3)	−0.0017 (3)	0.0017 (3)
O1	0.0169 (10)	0.0278 (12)	0.0539 (14)	0.0016 (9)	−0.0058 (9)	−0.0108 (10)
O2	0.0193 (10)	0.0245 (11)	0.0268 (10)	0.0047 (9)	−0.0032 (8)	−0.0011 (9)
O3	0.0185 (10)	0.0284 (11)	0.0216 (10)	−0.0023 (9)	0.0020 (8)	0.0012 (9)
C1	0.0259 (15)	0.0237 (15)	0.0185 (13)	−0.0004 (11)	0.0034 (10)	0.0011 (11)
C2	0.048 (2)	0.0244 (16)	0.0268 (16)	−0.0052 (14)	−0.0002 (13)	−0.0017 (12)
C3	0.0356 (18)	0.0297 (17)	0.0274 (15)	−0.0036 (14)	0.0041 (12)	−0.0068 (13)
C4	0.0422 (19)	0.0329 (17)	0.0201 (14)	−0.0031 (14)	−0.0001 (12)	−0.0044 (13)
C5	0.0207 (14)	0.0191 (14)	0.0207 (12)	−0.0003 (10)	0.0018 (10)	0.0008 (10)
C6	0.0217 (15)	0.0313 (16)	0.0324 (15)	−0.0026 (12)	0.0055 (11)	−0.0052 (12)
C7	0.0180 (13)	0.0208 (14)	0.0195 (13)	−0.0003 (10)	−0.0005 (9)	0.0018 (10)
C8	0.0183 (13)	0.0197 (13)	0.0173 (12)	0.0004 (10)	0.0007 (10)	0.0019 (10)
C9	0.0306 (16)	0.0290 (16)	0.0199 (13)	−0.0049 (13)	0.0037 (11)	0.0011 (12)
C10	0.0166 (13)	0.0201 (13)	0.0185 (12)	0.0023 (10)	0.0000 (9)	0.0030 (10)
C11	0.0174 (13)	0.0221 (14)	0.0278 (14)	0.0013 (10)	0.0006 (10)	−0.0006 (11)
C12	0.0240 (15)	0.0360 (19)	0.0398 (17)	0.0020 (13)	0.0077 (12)	−0.0019 (14)
C13	0.0262 (19)	0.064 (3)	0.064 (2)	−0.0159 (18)	0.0150 (17)	−0.010 (2)
C14	0.045 (2)	0.079 (3)	0.044 (2)	0.008 (2)	0.0183 (17)	0.018 (2)
C15	0.0236 (15)	0.0299 (17)	0.050 (2)	0.0006 (13)	−0.0059 (14)	0.0114 (15)
C16	0.044 (2)	0.0252 (18)	0.080 (3)	0.0043 (16)	−0.017 (2)	0.0063 (19)
C17	0.039 (2)	0.067 (3)	0.073 (3)	0.002 (2)	0.000 (2)	0.049 (2)
C18	0.0237 (14)	0.0247 (15)	0.0298 (15)	0.0001 (12)	0.0020 (11)	0.0003 (12)
C19	0.038 (2)	0.039 (2)	0.0381 (19)	0.0020 (16)	−0.0016 (14)	−0.0084 (15)
C20	0.0358 (18)	0.0216 (15)	0.0456 (19)	0.0034 (13)	−0.0023 (14)	0.0001 (14)
S3	0.0408 (4)	0.0261 (4)	0.0210 (3)	0.0026 (3)	0.0060 (3)	−0.0025 (3)
S4	0.0409 (4)	0.0210 (4)	0.0263 (4)	0.0013 (3)	−0.0093 (3)	−0.0033 (3)
Si2	0.0200 (4)	0.0211 (4)	0.0301 (4)	0.0008 (3)	−0.0004 (3)	0.0005 (3)
O4	0.0210 (10)	0.0232 (10)	0.0368 (11)	0.0011 (8)	−0.0046 (8)	0.0013 (9)
O5	0.0189 (10)	0.0244 (11)	0.0334 (11)	−0.0024 (9)	−0.0013 (8)	0.0020 (9)
O6	0.0175 (10)	0.0281 (11)	0.0238 (10)	0.0033 (8)	0.0006 (8)	−0.0012 (8)
C21	0.0246 (14)	0.0219 (14)	0.0169 (12)	0.0000 (11)	0.0025 (10)	−0.0034 (11)
C22	0.0375 (18)	0.0256 (15)	0.0280 (15)	0.0084 (13)	0.0012 (12)	0.0022 (12)
C23	0.0294 (16)	0.0298 (16)	0.0257 (15)	0.0018 (13)	0.0072 (12)	0.0044 (12)
C24	0.0434 (19)	0.0316 (17)	0.0198 (14)	0.0005 (14)	−0.0011 (12)	0.0033 (12)
C25	0.0205 (13)	0.0197 (14)	0.0214 (13)	0.0010 (11)	0.0006 (10)	−0.0015 (11)
C26	0.0220 (14)	0.0311 (16)	0.0330 (15)	0.0054 (13)	0.0063 (11)	0.0031 (13)
C27	0.0190 (13)	0.0203 (14)	0.0211 (13)	0.0006 (11)	−0.0008 (10)	−0.0046 (11)
C28	0.0203 (13)	0.0209 (14)	0.0187 (12)	0.0029 (11)	0.0010 (10)	−0.0021 (10)
C29	0.0395 (18)	0.0332 (17)	0.0199 (14)	0.0095 (14)	0.0030 (12)	−0.0034 (12)
C30	0.0175 (14)	0.0199 (14)	0.0209 (13)	0.0004 (10)	0.0014 (10)	−0.0021 (10)
C31	0.0205 (14)	0.0220 (13)	0.0259 (13)	−0.0005 (11)	−0.0001 (10)	0.0005 (11)
C32	0.0297 (17)	0.0385 (19)	0.0366 (17)	0.0037 (14)	0.0025 (13)	0.0103 (15)
C33	0.042 (2)	0.064 (3)	0.0306 (16)	−0.001 (2)	0.0052 (13)	0.0022 (18)
C34	0.036 (2)	0.038 (2)	0.055 (2)	−0.0006 (16)	0.0095 (16)	0.0163 (17)
C35	0.0280 (16)	0.0317 (17)	0.0405 (18)	−0.0092 (14)	0.0005 (13)	−0.0033 (14)
C36	0.037 (2)	0.0311 (19)	0.070 (3)	−0.0056 (16)	−0.0091 (18)	−0.0081 (18)
C37	0.0233 (18)	0.047 (2)	0.080 (3)	−0.0008 (16)	−0.0066 (17)	−0.010 (2)
C38	0.0318 (18)	0.039 (2)	0.047 (2)	−0.0044 (15)	0.0089 (14)	−0.0155 (16)
C39	0.048 (3)	0.050 (3)	0.087 (3)	0.008 (2)	0.019 (2)	−0.025 (2)
C40	0.058 (3)	0.081 (3)	0.040 (2)	−0.018 (2)	0.0149 (18)	−0.019 (2)

### Geometric parameters (Å, º)

**Table:**

S1—C2	1.816 (3)	S3—C24	1.813 (3)
S1—C1	1.819 (3)	S3—C21	1.814 (3)
S2—C1	1.814 (3)	S4—C22	1.813 (3)
S2—C4	1.818 (3)	S4—C21	1.815 (3)
Si1—O1	1.649 (2)	Si2—O4	1.649 (2)
Si1—C12	1.873 (3)	Si2—C35	1.877 (3)
Si1—C18	1.882 (3)	Si2—C32	1.882 (3)
Si1—C15	1.884 (3)	Si2—C38	1.883 (4)
O1—C11	1.408 (3)	O4—C31	1.420 (3)
O2—C10	1.439 (3)	O5—C30	1.440 (3)
O2—H100	0.76 (4)	O5—H103	0.80 (5)
O3—C7	1.445 (3)	O6—C27	1.448 (3)
O3—H101	0.66 (4)	O6—H102	0.76 (4)
C1—C5	1.540 (4)	C21—C25	1.544 (4)
C1—H1	1.0000	C21—H21	1.0000
C2—C3	1.518 (4)	C22—C23	1.520 (4)
C2—H2A	0.9900	C22—H22A	0.9900
C2—H2B	0.9900	C22—H22B	0.9900
C3—C4	1.511 (5)	C23—C24	1.523 (5)
C3—H3A	0.9900	C23—H23A	0.9900
C3—H3B	0.9900	C23—H23B	0.9900
C4—H4A	0.9900	C24—H24A	0.9900
C4—H4B	0.9900	C24—H24B	0.9900
C5—C7	1.532 (4)	C25—C26	1.531 (4)
C5—C6	1.533 (4)	C25—C27	1.537 (4)
C5—H5	1.0000	C25—H25	1.0000
C6—H6A	0.9800	C26—H26A	0.9800
C6—H6B	0.9800	C26—H26B	0.9800
C6—H6C	0.9800	C26—H26C	0.9800
C7—C8	1.536 (4)	C27—C28	1.529 (4)
C7—H7	1.0000	C27—H27	1.0000
C8—C10	1.531 (4)	C28—C30	1.531 (4)
C8—C9	1.533 (4)	C28—C29	1.539 (4)
C8—H8	1.0000	C28—H28	1.0000
C9—H9A	0.9800	C29—H29A	0.9800
C9—H9B	0.9800	C29—H29B	0.9800
C9—H9C	0.9800	C29—H29C	0.9800
C10—C11	1.520 (4)	C30—C31	1.518 (4)
C10—H10	1.0000	C30—H30	1.0000
C11—H11A	0.9900	C31—H31A	0.9900
C11—H11B	0.9900	C31—H31B	0.9900
C12—C13	1.522 (5)	C32—C33	1.547 (5)
C12—C14	1.533 (5)	C32—C34	1.547 (5)
C12—H12	1.0000	C32—H32	1.0000
C13—H13A	0.9800	C33—H33A	0.9800
C13—H13B	0.9800	C33—H33B	0.9800
C13—H13C	0.9800	C33—H33C	0.9800
C14—H14A	0.9800	C34—H34A	0.9800
C14—H14B	0.9800	C34—H34B	0.9800
C14—H14C	0.9800	C34—H34C	0.9800
C15—C16	1.531 (5)	C35—C37	1.533 (5)
C15—C17	1.536 (6)	C35—C36	1.538 (5)
C15—H15	1.0000	C35—H35	1.0000
C16—H16A	0.9800	C36—H36A	0.9800
C16—H16B	0.9800	C36—H36B	0.9800
C16—H16C	0.9800	C36—H36C	0.9800
C17—H17A	0.9800	C37—H37A	0.9800
C17—H17B	0.9800	C37—H37B	0.9800
C17—H17C	0.9800	C37—H37C	0.9800
C18—C20	1.535 (4)	C38—C40	1.533 (6)
C18—C19	1.536 (4)	C38—C39	1.546 (6)
C18—H18	1.0000	C38—H38	1.0000
C19—H19A	0.9800	C39—H39A	0.9800
C19—H19B	0.9800	C39—H39B	0.9800
C19—H19C	0.9800	C39—H39C	0.9800
C20—H20A	0.9800	C40—H40A	0.9800
C20—H20B	0.9800	C40—H40B	0.9800
C20—H20C	0.9800	C40—H40C	0.9800
			
C2—S1—C1	98.33 (15)	C24—S3—C21	98.88 (14)
C1—S2—C4	98.28 (14)	C22—S4—C21	98.81 (14)
O1—Si1—C12	104.60 (14)	O4—Si2—C35	103.93 (14)
O1—Si1—C18	110.06 (13)	O4—Si2—C32	110.38 (14)
C12—Si1—C18	115.97 (15)	C35—Si2—C32	112.94 (16)
O1—Si1—C15	106.58 (14)	O4—Si2—C38	107.91 (14)
C12—Si1—C15	109.22 (15)	C35—Si2—C38	111.46 (16)
C18—Si1—C15	109.91 (15)	C32—Si2—C38	109.95 (17)
C11—O1—Si1	132.1 (2)	C31—O4—Si2	128.14 (19)
C10—O2—H100	105 (3)	C30—O5—H103	104 (3)
C7—O3—H101	107 (3)	C27—O6—H102	108 (3)
C5—C1—S2	109.89 (19)	C25—C21—S3	110.08 (19)
C5—C1—S1	111.31 (19)	C25—C21—S4	111.50 (18)
S2—C1—S1	112.11 (16)	S3—C21—S4	112.18 (16)
C5—C1—H1	107.8	C25—C21—H21	107.6
S2—C1—H1	107.8	S3—C21—H21	107.6
S1—C1—H1	107.8	S4—C21—H21	107.6
C3—C2—S1	113.3 (2)	C23—C22—S4	113.6 (2)
C3—C2—H2A	108.9	C23—C22—H22A	108.8
S1—C2—H2A	108.9	S4—C22—H22A	108.8
C3—C2—H2B	108.9	C23—C22—H22B	108.8
S1—C2—H2B	108.9	S4—C22—H22B	108.8
H2A—C2—H2B	107.7	H22A—C22—H22B	107.7
C4—C3—C2	113.5 (3)	C22—C23—C24	113.1 (3)
C4—C3—H3A	108.9	C22—C23—H23A	109.0
C2—C3—H3A	108.9	C24—C23—H23A	109.0
C4—C3—H3B	108.9	C22—C23—H23B	109.0
C2—C3—H3B	108.9	C24—C23—H23B	109.0
H3A—C3—H3B	107.7	H23A—C23—H23B	107.8
C3—C4—S2	114.5 (2)	C23—C24—S3	114.9 (2)
C3—C4—H4A	108.6	C23—C24—H24A	108.5
S2—C4—H4A	108.6	S3—C24—H24A	108.5
C3—C4—H4B	108.6	C23—C24—H24B	108.5
S2—C4—H4B	108.6	S3—C24—H24B	108.5
H4A—C4—H4B	107.6	H24A—C24—H24B	107.5
C7—C5—C6	114.1 (2)	C26—C25—C27	113.7 (2)
C7—C5—C1	108.6 (2)	C26—C25—C21	112.3 (2)
C6—C5—C1	112.1 (2)	C27—C25—C21	108.7 (2)
C7—C5—H5	107.2	C26—C25—H25	107.2
C6—C5—H5	107.2	C27—C25—H25	107.2
C1—C5—H5	107.2	C21—C25—H25	107.2
C5—C6—H6A	109.5	C25—C26—H26A	109.5
C5—C6—H6B	109.5	C25—C26—H26B	109.5
H6A—C6—H6B	109.5	H26A—C26—H26B	109.5
C5—C6—H6C	109.5	C25—C26—H26C	109.5
H6A—C6—H6C	109.5	H26A—C26—H26C	109.5
H6B—C6—H6C	109.5	H26B—C26—H26C	109.5
O3—C7—C5	106.2 (2)	O6—C27—C28	110.3 (2)
O3—C7—C8	110.0 (2)	O6—C27—C25	105.9 (2)
C5—C7—C8	115.2 (2)	C28—C27—C25	116.2 (2)
O3—C7—H7	108.4	O6—C27—H27	108.1
C5—C7—H7	108.4	C28—C27—H27	108.1
C8—C7—H7	108.4	C25—C27—H27	108.1
C10—C8—C9	112.1 (2)	C27—C28—C30	112.4 (2)
C10—C8—C7	112.2 (2)	C27—C28—C29	110.3 (2)
C9—C8—C7	109.6 (2)	C30—C28—C29	112.4 (2)
C10—C8—H8	107.6	C27—C28—H28	107.1
C9—C8—H8	107.6	C30—C28—H28	107.1
C7—C8—H8	107.6	C29—C28—H28	107.1
C8—C9—H9A	109.5	C28—C29—H29A	109.5
C8—C9—H9B	109.5	C28—C29—H29B	109.5
H9A—C9—H9B	109.5	H29A—C29—H29B	109.5
C8—C9—H9C	109.5	C28—C29—H29C	109.5
H9A—C9—H9C	109.5	H29A—C29—H29C	109.5
H9B—C9—H9C	109.5	H29B—C29—H29C	109.5
O2—C10—C11	107.3 (2)	O5—C30—C31	106.5 (2)
O2—C10—C8	111.7 (2)	O5—C30—C28	112.3 (2)
C11—C10—C8	113.6 (2)	C31—C30—C28	113.8 (2)
O2—C10—H10	108.0	O5—C30—H30	108.0
C11—C10—H10	108.0	C31—C30—H30	108.0
C8—C10—H10	108.0	C28—C30—H30	108.0
O1—C11—C10	106.9 (2)	O4—C31—C30	108.2 (2)
O1—C11—H11A	110.3	O4—C31—H31A	110.1
C10—C11—H11A	110.3	C30—C31—H31A	110.1
O1—C11—H11B	110.3	O4—C31—H31B	110.1
C10—C11—H11B	110.3	C30—C31—H31B	110.1
H11A—C11—H11B	108.6	H31A—C31—H31B	108.4
C13—C12—C14	111.3 (3)	C33—C32—C34	110.8 (3)
C13—C12—Si1	114.6 (2)	C33—C32—Si2	111.1 (3)
C14—C12—Si1	114.3 (2)	C34—C32—Si2	112.3 (2)
C13—C12—H12	105.2	C33—C32—H32	107.4
C14—C12—H12	105.2	C34—C32—H32	107.4
Si1—C12—H12	105.2	Si2—C32—H32	107.4
C12—C13—H13A	109.5	C32—C33—H33A	109.5
C12—C13—H13B	109.5	C32—C33—H33B	109.5
H13A—C13—H13B	109.5	H33A—C33—H33B	109.5
C12—C13—H13C	109.5	C32—C33—H33C	109.5
H13A—C13—H13C	109.5	H33A—C33—H33C	109.5
H13B—C13—H13C	109.5	H33B—C33—H33C	109.5
C12—C14—H14A	109.5	C32—C34—H34A	109.5
C12—C14—H14B	109.5	C32—C34—H34B	109.5
H14A—C14—H14B	109.5	H34A—C34—H34B	109.5
C12—C14—H14C	109.5	C32—C34—H34C	109.5
H14A—C14—H14C	109.5	H34A—C34—H34C	109.5
H14B—C14—H14C	109.5	H34B—C34—H34C	109.5
C16—C15—C17	111.4 (3)	C37—C35—C36	109.8 (3)
C16—C15—Si1	110.8 (3)	C37—C35—Si2	113.3 (3)
C17—C15—Si1	111.5 (2)	C36—C35—Si2	113.9 (3)
C16—C15—H15	107.6	C37—C35—H35	106.4
C17—C15—H15	107.6	C36—C35—H35	106.4
Si1—C15—H15	107.6	Si2—C35—H35	106.4
C15—C16—H16A	109.5	C35—C36—H36A	109.5
C15—C16—H16B	109.5	C35—C36—H36B	109.5
H16A—C16—H16B	109.5	H36A—C36—H36B	109.5
C15—C16—H16C	109.5	C35—C36—H36C	109.5
H16A—C16—H16C	109.5	H36A—C36—H36C	109.5
H16B—C16—H16C	109.5	H36B—C36—H36C	109.5
C15—C17—H17A	109.5	C35—C37—H37A	109.5
C15—C17—H17B	109.5	C35—C37—H37B	109.5
H17A—C17—H17B	109.5	H37A—C37—H37B	109.5
C15—C17—H17C	109.5	C35—C37—H37C	109.5
H17A—C17—H17C	109.5	H37A—C37—H37C	109.5
H17B—C17—H17C	109.5	H37B—C37—H37C	109.5
C20—C18—C19	109.4 (3)	C40—C38—C39	110.9 (4)
C20—C18—Si1	114.1 (2)	C40—C38—Si2	112.7 (3)
C19—C18—Si1	114.0 (2)	C39—C38—Si2	113.4 (3)
C20—C18—H18	106.2	C40—C38—H38	106.4
C19—C18—H18	106.2	C39—C38—H38	106.4
Si1—C18—H18	106.2	Si2—C38—H38	106.4
C18—C19—H19A	109.5	C38—C39—H39A	109.5
C18—C19—H19B	109.5	C38—C39—H39B	109.5
H19A—C19—H19B	109.5	H39A—C39—H39B	109.5
C18—C19—H19C	109.5	C38—C39—H39C	109.5
H19A—C19—H19C	109.5	H39A—C39—H39C	109.5
H19B—C19—H19C	109.5	H39B—C39—H39C	109.5
C18—C20—H20A	109.5	C38—C40—H40A	109.5
C18—C20—H20B	109.5	C38—C40—H40B	109.5
H20A—C20—H20B	109.5	H40A—C40—H40B	109.5
C18—C20—H20C	109.5	C38—C40—H40C	109.5
H20A—C20—H20C	109.5	H40A—C40—H40C	109.5
H20B—C20—H20C	109.5	H40B—C40—H40C	109.5
			
C12—Si1—O1—C11	−128.0 (3)	C35—Si2—O4—C31	−178.8 (2)
C18—Si1—O1—C11	−2.8 (3)	C32—Si2—O4—C31	59.8 (3)
C15—Si1—O1—C11	116.4 (3)	C38—Si2—O4—C31	−60.4 (3)
C4—S2—C1—C5	−173.4 (2)	C24—S3—C21—C25	−173.8 (2)
C4—S2—C1—S1	62.28 (19)	C24—S3—C21—S4	61.44 (19)
C2—S1—C1—C5	173.0 (2)	C22—S4—C21—C25	173.3 (2)
C2—S1—C1—S2	−63.40 (19)	C22—S4—C21—S3	−62.73 (19)
C1—S1—C2—C3	61.1 (3)	C21—S4—C22—C23	61.1 (3)
S1—C2—C3—C4	−65.9 (4)	S4—C22—C23—C24	−65.4 (3)
C2—C3—C4—S2	65.3 (4)	C22—C23—C24—S3	64.5 (3)
C1—S2—C4—C3	−59.6 (3)	C21—S3—C24—C23	−58.9 (3)
S2—C1—C5—C7	158.25 (18)	S3—C21—C25—C26	−73.4 (3)
S1—C1—C5—C7	−77.0 (2)	S4—C21—C25—C26	51.8 (3)
S2—C1—C5—C6	−74.8 (3)	S3—C21—C25—C27	159.84 (18)
S1—C1—C5—C6	50.0 (3)	S4—C21—C25—C27	−75.0 (2)
C6—C5—C7—O3	168.4 (2)	C26—C25—C27—O6	168.7 (2)
C1—C5—C7—O3	−65.7 (3)	C21—C25—C27—O6	−65.3 (3)
C6—C5—C7—C8	46.4 (3)	C26—C25—C27—C28	45.9 (3)
C1—C5—C7—C8	172.3 (2)	C21—C25—C27—C28	171.9 (2)
O3—C7—C8—C10	−62.8 (3)	O6—C27—C28—C30	−59.3 (3)
C5—C7—C8—C10	57.2 (3)	C25—C27—C28—C30	61.1 (3)
O3—C7—C8—C9	62.5 (3)	O6—C27—C28—C29	67.0 (3)
C5—C7—C8—C9	−177.5 (2)	C25—C27—C28—C29	−172.6 (2)
C9—C8—C10—O2	−61.4 (3)	C27—C28—C30—O5	62.6 (3)
C7—C8—C10—O2	62.4 (3)	C29—C28—C30—O5	−62.6 (3)
C9—C8—C10—C11	60.2 (3)	C27—C28—C30—C31	−176.3 (2)
C7—C8—C10—C11	−176.0 (2)	C29—C28—C30—C31	58.5 (3)
Si1—O1—C11—C10	177.0 (2)	Si2—O4—C31—C30	149.5 (2)
O2—C10—C11—O1	−172.9 (2)	O5—C30—C31—O4	178.0 (2)
C8—C10—C11—O1	63.0 (3)	C28—C30—C31—O4	53.7 (3)
O1—Si1—C12—C13	−178.7 (3)	O4—Si2—C32—C33	52.6 (3)
C18—Si1—C12—C13	59.9 (3)	C35—Si2—C32—C33	−63.3 (3)
C15—Si1—C12—C13	−64.9 (3)	C38—Si2—C32—C33	171.5 (2)
O1—Si1—C12—C14	51.1 (3)	O4—Si2—C32—C34	−72.2 (3)
C18—Si1—C12—C14	−70.3 (3)	C35—Si2—C32—C34	171.9 (3)
C15—Si1—C12—C14	164.9 (3)	C38—Si2—C32—C34	46.7 (3)
O1—Si1—C15—C16	61.1 (3)	O4—Si2—C35—C37	−161.4 (3)
C12—Si1—C15—C16	−51.4 (3)	C32—Si2—C35—C37	−41.7 (3)
C18—Si1—C15—C16	−179.6 (2)	C38—Si2—C35—C37	82.6 (3)
O1—Si1—C15—C17	−63.6 (3)	O4—Si2—C35—C36	−35.0 (3)
C12—Si1—C15—C17	−176.1 (3)	C32—Si2—C35—C36	84.7 (3)
C18—Si1—C15—C17	55.6 (3)	C38—Si2—C35—C36	−150.9 (3)
O1—Si1—C18—C20	−71.4 (3)	O4—Si2—C38—C40	−59.9 (3)
C12—Si1—C18—C20	47.1 (3)	C35—Si2—C38—C40	53.6 (3)
C15—Si1—C18—C20	171.5 (2)	C32—Si2—C38—C40	179.6 (3)
O1—Si1—C18—C19	161.9 (2)	O4—Si2—C38—C39	173.1 (3)
C12—Si1—C18—C19	−79.6 (3)	C35—Si2—C38—C39	−73.4 (3)
C15—Si1—C18—C19	44.8 (3)	C32—Si2—C38—C39	52.6 (3)

### Hydrogen-bond geometry (Å, º)

**Table:**

*D*—H···*A*	*D*—H	H···*A*	*D*···*A*	*D*—H···*A*
O2—H100···O3	0.76 (4)	2.00 (4)	2.705 (4)	154 (5)
O5—H103···O6	0.81 (5)	1.94 (5)	2.689 (3)	153 (5)
O3—H101···O5^i^	0.66 (3)	2.11 (4)	2.751 (3)	167 (5)
O6—H102···O2^ii^	0.76 (4)	1.93 (4)	2.685 (3)	175 (4)

Symmetry codes: (i) *x*−1, *y*, *z*; (ii) *x*+1, *y*−1, *z*.
