# Supplementary figures and images for: Crystal structure of (−)-(2R,3S,4R,5R)-5-(1,3-di­thian-2-yl)-3-methyl-1-(triiso­propyl­sil­yloxy)hexane-2,4-diol
Source: Acta Crystallogr Sect E Struct Rep Online. 2014 Nov 21;70(Pt 12):o1285–6. doi: 10.1107/S160053681402443X (PMC4257393; doi:10.1107/S160053681402443X)

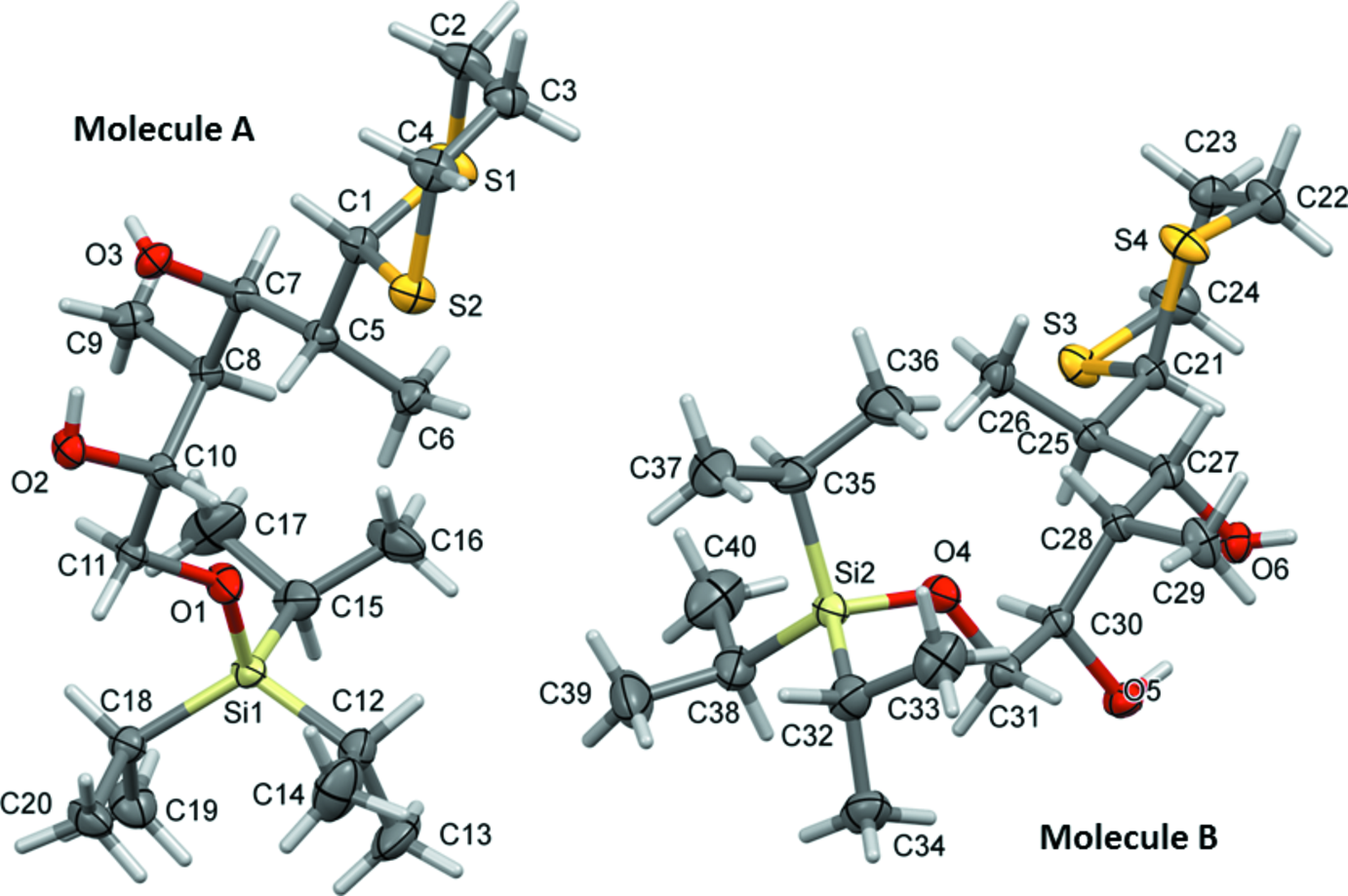

Supplement: Supplementary file 4 [file e-70-o1285-fig1.tif]

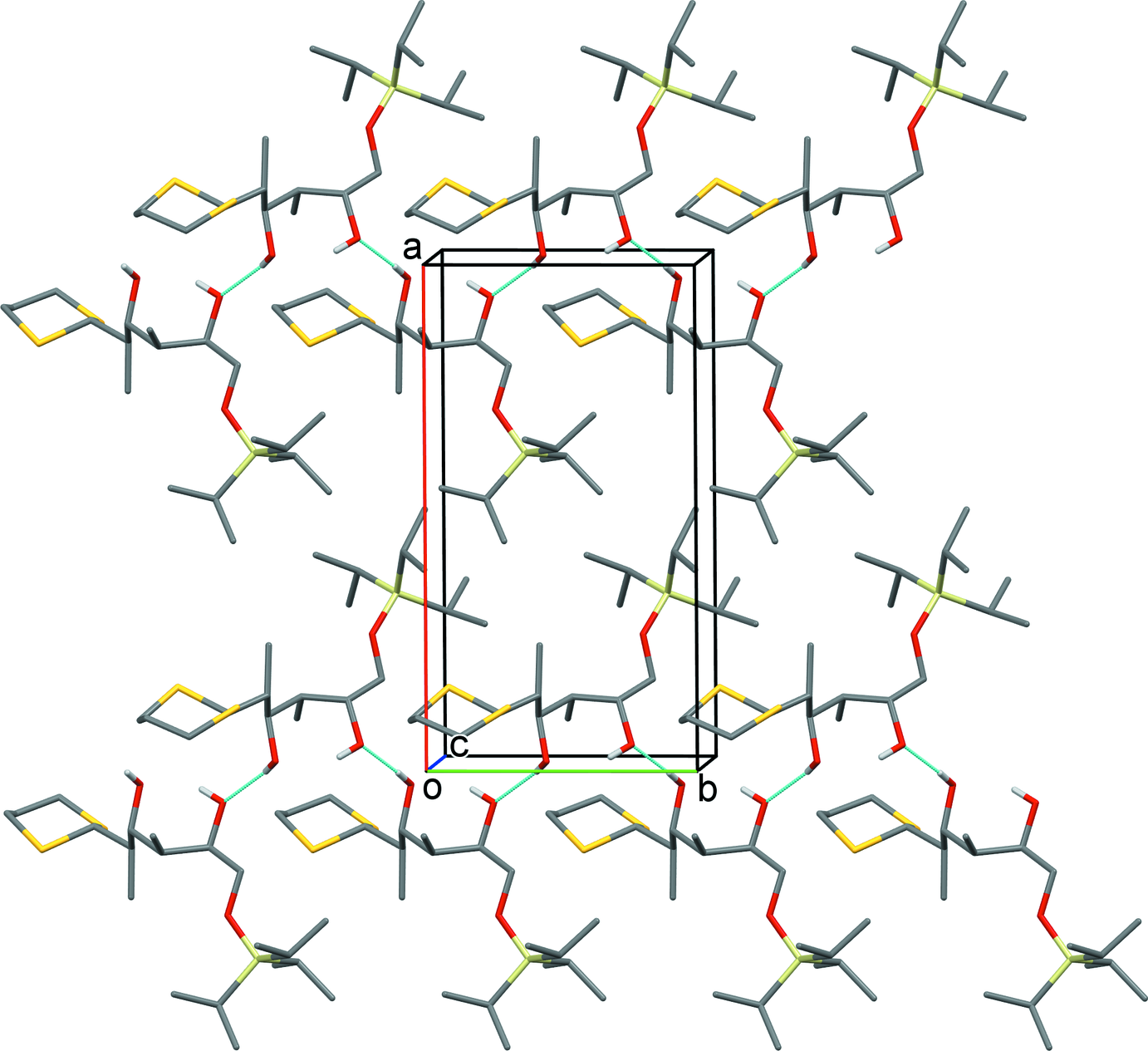

Supplement: Supplementary file 5 [file e-70-o1285-fig2.tif]

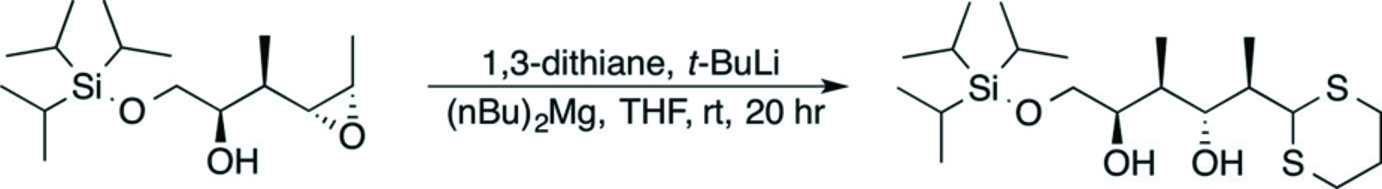

Supplement: Supplementary file 6 [file e-70-o1285-fig3.tif]
